# Artificial Intelligence in digital pathology of breast cancer, new era of practice?

**DOI:** 10.1097/JS9.0000000000002953

**Published:** 2025-07-08

**Authors:** Wenjing Li, Sijing Ye, Zimeng Jin, Lin Chen, Yuqing Chao, Guikang Wei, Qinyi Huang, Huakang Tu, Qinchuan Wang

**Affiliations:** a Department of Surgical Oncology, Affiliated Sir Run Run Shaw Hospital, Zhejiang University School of Medicine, Zhejiang, China; b School of Public Health, Zhejiang University School of Medicine, Hangzhou, Zhejiang, China

**Keywords:** artificial intelligence, biomarkers, breast cancer, digital pathology

## Abstract

Breast cancer is the most common cancers among women worldwide. Early diagnosis and personalized medicine are crucial for the treatment of breast cancer. With the development of computer science and the emergence of whole slide imaging technology, artificial intelligence (AI) is having a surprisingly positive impact on the field of pathology, including breast pathology. The deployment of AI provides powerful tools for research in digital pathology and provides potential solutions in precision medicine in breast cancer. In this review, we systematically reviewed the applications of AI in digital pathology of breast cancer, including the identification of histological features, such as tumor-infiltrating lymphocytes, and the evaluation of classical biomarkers, such as human epidermal growth factor receptor 2, estrogen receptor, progesterone receptor. We also introduce the combined use of AI with multi-omics data in outcome prediction and treatment in breast cancer, and outline the evolution of AI methods applied in digital pathology. Collectively, the robustly evolving AI technologies would profoundly impact the precision pathology and medicine in breast cancer.

## Introduction

Breast cancer is one of the most common cancers worldwide, accounting for approximately 30% of cancers in women, and it is also one of the leading causes of cancer-related deaths among women globally^[1]^. Early diagnosis of breast cancer is crucial for the treatment and prognosis of patients. Currently, pathological examination remains the gold standard for breast cancer diagnosis^[2]^. However, traditional histopathological examination is very time-consuming and requiring trained pathologists, which significantly restrained the precision diagnosis of breast cancer^[3]^. Also, discrepancies also exist in the diagnosis among different pathologists or laboratories for specific breast neoplasms like lobular neoplasia^[4]^. Therefore, novel methodologies are urgently required for breast cancer histological evaluation.

**HIGHLIGHTS**

- Artificial intelligence (AI) could significantly empower the identification of histological features of breast cancer.
- AI could potentially replace classical evaluation of biomarkers in breast cancer.
- AI could conjugate with multi-omics data in the precision medicine of breast cancer.
- AI promote the evolution of methodologies in digital pathology of breast cancer.

**Figure 1.**
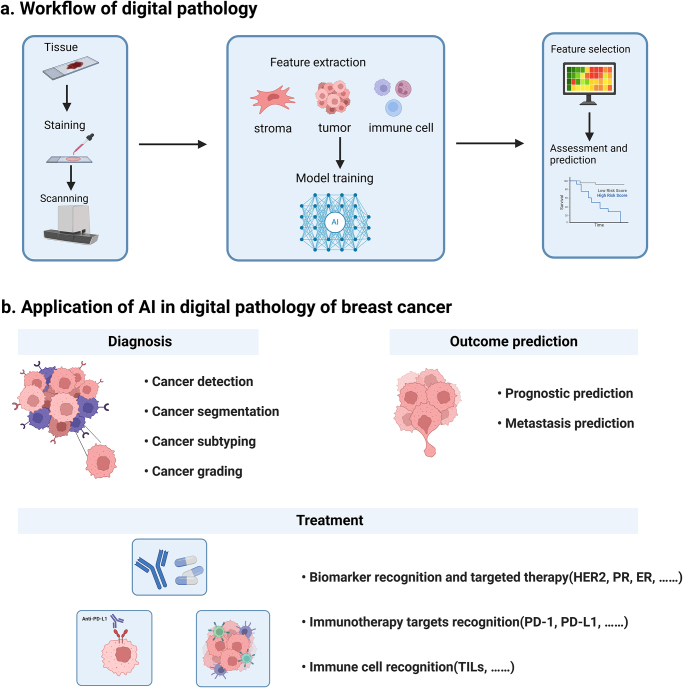
Application of artificial intelligence in digital pathology of breast cancer. (A) The basic workflow of digital pathology. (B) The application of AI in digital pathology of breast cancer. AI has been applied in multiple aspects in breast cancer, including the diagnosis, outcome prediction, and treatment.

With the development of computer science and the emergence of whole slide imaging (WSI) technology, digital pathology has gradually risen and begun to play a role in clinical practice. Digital pathology utilizes whole slide scanners to convert histopathological slides into high-resolution digital images, known as WSI, and then analyzes these digitalized images through automated computer analysis techniques^[5]^ (Fig. 1a). Compared to traditional microscopy, WSI images offer higher resolution, easier storage and transmission. Currently, multiple digital pathology datasets have been generated, enabling the application of artificial intelligence (AI) in interpretation of those slides. The development of digital pathology has made it possible to mine previously undeveloped features from conventional cancer histopathological images, thereby providing potential diagnostic, prognostic, predictive, and other clinical relevant information^[6]^.

AI has been developing rapidly, providing powerful tools for research in digital pathology. AI refers to the ability of machines to simulate human intelligence in performing tasks involving decision-making and problem-solving. With the advancements of algorithms, improvements in computing power, and the establishment of more available datasets, AI has been widely applied in multiple fields. Currently, the most commonly used AI techniques in medical research is machine learning (ML). ML-based methods can learn the patterns and regularities from the input data, and make decisions on new data without human instruction. ML mainly consists of supervised learning, and unsupervised learning. Supervised learning uses labeled data for training, while unsupervised learning focuses on extracting patterns and structures from unlabeled data. Deep learning (DL) is a specialized method of ML developed based on neural networks. This approach mimics the working method of human brain by constructing and training multi-layer neural networks to process and interpret complex data. A representative application of deep learning is image recognition and classification, including medical imaging^[7]^. In recent years, DL has gradually been the mainstream in the field of digital pathology research. It has been applied to a range of image processing and classification tasks, demonstrating exceptional performance in both low-level tasks related to object recognition (such as detection and segmentation) and high-level tasks (such as predicting disease diagnosis and treatment response based on pathological images)^[8]^. Meanwhile, as the use of AI in research and the related literatures are increasing, we strictly followed the TITAN Guideline 2025 to increase the transparency of the review^[9]^. The completed TITAN Guideline checklist is shown in Supplementary Digital Content Table 1, available at: http://links.lww.com/JS9/E624

AI has been applied in various aspects of digital pathology for breast cancer, including the identification of tumor components^[10]^, classification of subtypes^[11]^, grading of histology^[12]^, and the prediction of disease outcomes^[13,14]^ (shown in Fig. 1b and Table 1). In addition, AI-based methods also demonstrated exceptional performance in the assessment of multiple breast cancer biomarkers, including human epidermal growth factor receptor 2 (HER2)^[15]^, estrogen receptor (ER), progesterone receptor (PR)^[16]^, and Ki67^[17]^. Furthermore, AI can also be used for the identification and counting of immune cells, including tumor-infiltrating lymphocytes (TILs)^[18]^, cell mitosis^[19]^, and others. Overall, AI can be integrated into diverse areas of the clinical workflow for breast cancer, such as screening, diagnosis, staging, treatment, and prognosis.

**Table 1:** Overview of the application of AI in digital pathology of breast cancer

Reference	Task	Staining	Method	Dataset	Result
^[12]^	Grading	H&E	DL	1567 patients from four different studies	DeepGrade provides independent prognostic information (HR = 2.94)
^[11]^	Classification	H&E	Class structure-based deep convolutional neural network (CSDCNN)	7,909 images from the dataset BreaKHis	The average accuracy was 93.2%
^[20]^	Classification	IHC	DL	600 slides from Kasturba Medical College	Consistent with the pathologist’s findings
^[13]^	Prognostic prediction	H&E and multiplex IHC	IMage-based Pathological REgistration and Segmentation Statistics (IMPRESS)	126 patients who had underwent surgery after NAC	HER2 + AUC = 0.8975; TNBC AUC = 0.7674
^[21]^	Prognostic prediction	H&E	Open-source software, QuPath, and CNN	920 TNBC patients included five independent cohorts	TILs variables had significant prognostic association with outcomes (*P* ≤ 0.01 for all comparisons)
^[22]^	Prognostic prediction	H&E	DL	262 eligible patients operated at two specific hospitals	HR: 0.81, 95% CI: 0.72–0.92, *P* = 0.001
^[23]^	Prognostic prediction	H&E and IHC	DL	A large prospective cohort from CPS-II and three independent cohorts	HiPS is a robustly validated biomarker
^[24]^	Prognostic prediction	H&E	DL	Two multi-centric cohorts: AUBC (n = 318) and TCGA (n = 111)	Digi-sTILs and Digi-TAS scores giving C-index values of 0.65 (*P* = 0.0189) and 0.60 (*P* = 0.0437)
^[25]^	Prognostic prediction	H&E	A hierarchical self-attention-guided deep learning framework	207 patients treated with NAC followed by surgery	AUC = 0.89 and F1-score = 90% for predicting pCR
^[14]^	Metastasis	H&E	DL	399 slides from Camelyon16 challenge, and 108 slides from the separate dataset (DS2)	The AUC was 99% and 99.6% respectively on the two test sets

Overview of the application of AI in digital pathology of breast cancer.

AUBC, The Australian Breast Cancer Tissue Bank; AUC, area under curve; CNN, convolutional Neural Networks; CPS-II, the Cancer Prevention Study-II; DL, deep learning; H&E, hematoxylin and eosin; HER2, human epidermal growth factor receptor 2; HR, hazard ratio; IHC, immunohistochemistry; NAC, neoadjuvant chemotherapy; pCR, pathological complete response; TILs, tumor-infiltrating lymphocytes; TNBC, triple-negative breast cancer.

In this review, we systematically summarized the existing literatures regarding the application of AI in digital pathology for breast cancer (Supplementary Digital Content Figure 1, available at: http://links.lww.com/JS9/E624). First, we discuss the achievements of AI in the identification of histological features and classical biomarkers of breast cancer. Second, we summarize the feasibility and advantage of conjugating AI with other multi-omics data to predict the prognosis of breast cancer. Finally, we briefly outline the evolution of methodologies of AI used in digital pathology research of breast cancer.

## Histological features as biomarkers predicting outcomes of breast cancer

### AI in TIL recognition

TILs are important prognostic and predictive biomarkers in triple-negative breast cancer (TNBC) and HER2-positive breast cancer, as well as many other types of solid tumors. Assessing TILs has significant clinical implications in breast cancer^[18,26,27]^. A detailed standardized system has been established for visual TILs assessment on hematoxylin and eosin (H&E)-stained slides in breast cancer^[28]^. The scoring is a semi-quantitative assessment that determines the percentage of infiltrated lymphocytes on the defined areas of stroma or tumor visible on the slide (average TIL%). Although some studies have demonstrated the effectiveness of this assessment method^[29]^, differences among individuals, limitations in accuracy, intra-tumoral heterogeneity, the spatial distribution of TILs and other factors still lead to variability between observers and the lack of ability to capture details about the distribution of immune cells within the specimen. In response to these challenges, many analytical methods utilizing AI for the automatic identification and scoring of TILs have emerged as promising approaches (Table 2).

**Table 2:** The application of AI in TILs recognition of breast cancer

Reference	Task	Staining	Method	Dataset	Result
^[30]^	TILs assessment	H&E	Deep neural networks	An Asian (n = 184) and a Caucasian (n = 117) cohort	The automatic TIL (aTIL) score was associated with disease free survival (DFS) (HR = 0.96)
^[21]^	TIL scoring in TNBC	H&E	Open-source software, QuPath, and CNN	920 TNBC patients included five independent cohorts	TIL variables had significant prognostic association with outcomes (*P* ≤ 0.01 for all comparisons)
^[22]^	Quantify stromal TILs density in TNBC	H&E	CNN	262 eligible patients operated at two specific hospitals	Establishing a quantitative and interpretable score (HR: 0.81, 95% CI: 0.72–0.92, *P* = 0.001)
^[31]^	Spatial TILs analysis to predict pCR after NAC in TNBC	H&E	Lunit SCOPE IO, an AI-powered H&E Whole-Slide Image (WSI) analyzer	954 patients treated with NAC in two centers in Korea	iTIL score was an independent predictor of pCR (adjusted odds ratio 1.211 per 1 point (%) change in the score, *P* < 0.001).
^[32]^	Assess the abundance and spatial distribution of TILs	H&E	CNN (VGG16, Inception-V4, and ResNet-34)	Patches from 23 TCGA cancer types	The new TIL models all achieve better performance with improvements of up to 13% in accuracy and 15% in F-score.
^[33]^	Generate combined maps of cancer regions and TILs	H&E	CNN (34-layer ResNet, 16-layer VGG, and Inception v4)	198 WSIs from cancer registry system and 1090 WSIs from TCGA	The results compared favorably with those obtained by using the best published methods.
^[35]^	Automatic detection of lymphocytes	IHC	DL	83 slides of 3 cancer types from nine different medical centers	U-Net obtained an F1-score of 0.78 and the highest agreement with manual evaluation (κ= 0.72).

Some researches about the application of AI in TILs recognition of breast cancer are listed in this table.

CNN, convolutional neural networks; DL, deep learning; H&E, hematoxylin and eosin; HR, hazard ratio; IHC, immunohistochemistry; NAC, neoadjuvant chemotherapy; pCR, pathological complete response; TILs, tumor-infiltrating lymphocytes; TNBC, triple-negative breast cancer; VGG,Visual Geometry Group; WSIs, whole slide images.

Peng Sun *et al*^[30]^ established a deep learning-based method for computational TILs assessment (CTA), although it still relies on regions manually annotated by pathologists. They employed a cell-based approach and trained three deep neural networks specifically for nuclear segmentation, nuclear classification, and necrosis classification. Subsequently, all cell nuclei in non-necrotic invasive carcinoma area were segmented and classified, and a cell classification algorithm was used to identify regions dense with tumor, stroma, and lymphocytes. The study found that automatic TILs (aTILs) scoring is consistent with manual TILs (mTILs) scoring, and the combined model of aTILs and mTILs could assist pathologists in performing risk management and decision-making tasks. Bai *et al*^[21]^ proposed an algorithm using the open-source software QuPath to establish a neural network classifier for tumor cells, lymphocytes, fibroblasts, and other cells on H&E-stained sections. The algorithm outputs five quantitative variables of prognostic significance, including the total area of TILs in the stroma (percentage); number of TILs, stromal cells, the total number of cells in annotated area, and the proportional number of TILs relative to the tumor. However, the inclusion of tumor regions and the exclusion of non-invasive epithelium were still manually annotated by experienced pathologists. Thagaard *et al*^[22]^ reported a fully automated digital image analysis pipeline using Visiopharm platform (Visiopharm A/S, Hørsholm, Denmark), in which a tissue-level model identifies tissue types and then automatically recognizes invasive tumors, non-invasive breast structures, stroma, and necrotic areas without manual interaction. Subsequently, a cell-level model identifies stromal TILs and reports the density of stromal TILs as a quantitative variable. This method for assessing stromal TILs follows all complex aspects of the guidelines set by the International Immuno-Oncology Biomarker Working Group (TIL-WG).

There are also studies proposing methods based on the spatial distribution of TILs^[30–32]^. For example, Han Le *et al*^[33]^, generated a combined maps of cancer regions and TILs in WSIs of breast cancer for routine diagnosis based on CNN analysis pipeline. The merged maps provide insights into the structural patterns and spatial distribution of lymphocyte infiltration, facilitating the quantification of TILs. In addition, some studies have used staining agents other than H&E staining, such as multi-panel immunofluorescence^[34]^ and immunohistochemistry (IHC)^[35]^.

However, there are still some defects in the current methods of TIL identification based on AI. The most frequent cause of inconsistent TILs assessment results compared to manual scoring is the inclusion of incorrectly assessed areas, such as necrotic areas^[36]^. Other reasons may include technical factors that influence ML algorithms, slide-related issues, heterogeneity in TILs distribution, and others. Therefore, many technological and procedural standardizations are necessary to counteract model-decay and interinstitutional differences in workflow. Public frameworks or infrastructures to work collaboratively on different labeling strategies need to be established to ensure that AI algorithms can identify and process all histological components, including ductal carcinoma in situ (DCIS), fibrosis, hyalinization, and granulocytes^[36]^. And collaborative community-driven projects to create generalizable AI algorithms are also essential.

### AI applications in other immune cells recognition in breast cancer

There are millions of immune cells in the tumor microenvironment (TME) of breast cancer, and the internal heterogeneity of these immune cells may explain the distinct responses to immunotherapy^[37]^. Therefore, identifying different types of immune cells may be a key step in understanding the complex interactions of anti-tumor immunity. A study integrated contrastive learning and weakly supervised learning to identify tumor-associated macrophages (TAMs) and the potential benefits of immunotherapy in breast cancer^[38]^. They found that the computational pathological features could accurately predict the proportion of tumor-infiltrating immune cells, particularly the level of macrophage infiltration. This study used self-supervised contrastive learning to extract informative features, without the need for manual annotation and can capture tile-level features more effectively. In another study, Mieke *et al*^[39]^ performed IHC staining the pan-macrophage marker CD68 and the M2-like macrophage markers CD163, CSF-1 R, and CD206 on a set of tissue microarrays of breast cancer. They quantified the number of TAMs using the digital image analysis (DIA) algorithm, and found that macrophages and their subsets were significantly associated with unfavorable clinicopathological characteristics in Luminal B subtype of breast cancer patients. This study included the large breast cancer series with a long-term follow-up and availability of well-characterized clinical data. But the breast cancer subtype groups are small and other immune microenvironment markers were not considered. Also, Chang Gong *et al*^[40]^ developed one method to detect CD8^+^ T cells from a set of whole IHC slides, quantifying spatial heterogeneity of TME using multiple metrics through spatial point pattern analysis and morphometric analysis. The results indicated that the number of high-density T cell clusters were higher in both circular and elongated shapes in patients who responded to PD-1 blockade therapy. A limitation of this study is the small number of patients involved (n = 29), but the methods used can be applied to larger patient cohorts. However, studies regarding the recognition of other immune cells are relatively limited. The identification of TILs remains the major research trend of the assessment of immune cells employing AI in digital pathology.

### AI identified histo-biomarkers as signature for breast cancer

TME is crucial for the occurrence, development, and treatment of cancer. Cancer progression involves a range of biochemical mechanisms, including cancer-permissive inflammation, activation of repair cascade responses, immune modulation, hypoxia-driven metabolic disorders, and epithelial-to-mesenchymal transition, and others^[41]^. Many of these characteristics are reflected in changes in the density, appearance, and spatial clustering of cancer-associated fibroblasts, TILs, and the stromal matrix^[42]^. Assessing these histological features could establish prognostic signatures for breast cancer, such as the Nottingham histological grade^[43]^. However, many potential features in histopathological images could not be steadily captured and evaluated. Therefore, utilizing AI to establish novel histo-biomarkers for comprehensive assessment of histological characteristics of breast cancer is a trend warrants further exploring.

Mohamed *et al*^[23]^ designed the Histomic Prognostic Signature (HiPS) that aligns with American Joint Committee on Cancer staging. This signature integrates current methods available: slides stained with H&E as well as ER, PR, and HER2 panels. HiPS comprehensively assesses the entire tumor microenvironment, including non-tumor factors, and employs DL to accurately map cellular and tissue structures to measure epithelial, stromal, immune, and spatial interaction characteristics. The results indicated that HiPS consistently outperformed pathologists in predicting survival outcomes, independent of tumor-node-metastasis stage and pertinent variables. But this study described each tissue region and cell nucleus by a set of morphological and spatial features, which results in the loss of potentially useful information, such as the outsized influence of small foci of angioinvasion. Another study employed deep learning algorithms to develop a set of AI-based features for digital spatial tumor microenvironment (sTME) and explored their prognostic value in TNBC^[24]^. The study utilized standard H&E histological images of TNBC, which segmenting tissue regions into tumor, stroma, and lymphocytes, and calculating quantitative features of their spatial relationships. They also established the digital stromal tumor-infiltrating lymphocytes (Digi-sTILs) score and the digital tumor-associated stroma (Digi-TAS) score, indicating that these scores were significant prognostic factors for predicting breast cancer-specific survival. This study is the first to show the importance of stromal features in TNBC survival outcomes with automatic quantification based on a deep learning approach. But additional analyses using larger patient cohorts are still needed to confirm these findings.

In summary, histo-biomarkers identified by AI could comprehensively evaluate the characteristics of the tumor microenvironment, which could significantly enhance the interpretation of TME in breast cancer as well as the management of breast cancer patients.

## Could AI replace classical evaluation of biomarkers in breast cancer?

### AI in evaluation of ER, PR, HER2, Ki67 expression

The detection of biomarkers like ER, PR, HER2, and Ki67 in breast cancer is crucial for the diagnosis and treatment of the disease^[44]^. In routine clinical practice, pathologists visually analyse biopsy tissue sections under a microscope. This visual examination is often subjective and prone to error. Evaluating biomarkers using AI can not only improve diagnostic consistency and accuracy, but can also help saving manpower and reducing costs. At the same time, repeatable automated processes can greatly increase the speed of diagnosis. Therefore, incorporating AI tools to evaluate these biomarkers could be objective, repeatable and cost-effective.

#### HER2

HER2 is one of the most important prognostic and therapeutic biomarkers in breast cancer. Up to 20% of breast cancers exhibit HER2 protein overexpression or gene amplification^[45]^. With the development of the new generation of antibody drug conjugates (ADCs) targeting HER2, precise HER2 evaluation is crucial for HER2-targeted treatment strategies. IHC, either alone or in combination with in situ hybridization (ISH), is the standard method to determine HER2 expression. According to the guidelines, the IHC expression pattern is scored as negative (0 or 1 +), equivocal (2 +), or positive (3 +). Equivocal cases require ISH to clarify the HER2 gene amplification status.

Currently, AI has been widely applied to the evaluation of HER2 expression levels in IHC images, which has achieved identical accuracy compare to human pathologists (Table 3). Monjoy Saha *et al*^[46]^ proposed a deep learning-based HER2 deep neural network (Her2Net) for the identification, segmentation, and classification of cell membranes and nuclei in HER2-stained IHC images, requiring minimal user intervention. The proposed Her2Net were proven to have high accuracy and broad applicability in HER2 scoring of breast cancer, and has been used as a patch-based segmentation, classification, and scoring tool. Manh-Dan *et al*^[15]^ proposed a semi-automated, two-stage deep learning algorithm to spatially classify HER2 into four classes. The produced spatial HER2 classification maps provide support for HER2 slides that are difficult to score. Another study proposed a new model based on Deep Reinforcement Learning (DRL), which learns to identify certain diagnosis-related Region of Interest (ROI) by following a parameterized strategy^[47]^. The selected ROI is processed using recurrent and residual convolutional networks to learn discriminative features for different HER2 scores and predict the next position, waiving the process of whole sub-image patches. This study demonstrated that the proposed model outperforms other methods based on state-of-the-art deep convolutional networks. However, a challenge remains in that some times the model selects regions that may not appear to be diagnostically relevant due to tumor heterogeneity.

**Table 3:** The application of AI in HER2 assessment of breast cancer

Reference	Task	Staining	Method	Dataset	Result
^[46]^	HER2 scoring, cell membrane and nucleus detection, segmentation, and classification	IHC	DL	79 monoclonal antibody-stained WSIs from the online database of University of Warwick	Her2Net achieved 96.64% precision, 98.33% accuracy, 96.71% F-score, 93.08% negative predictive value, and 6.84% false-positive rate.
^[15]^	HER2 scoring and classification	IHC	A semi-automatic, two-stage, weakly supervised DL approach	370 WSIs from hospitals, contest training set, and AIDPATH database	The method achieved a performance of 0.78 in F1-score on the test set
^[47]^	Automated HER2 scoring	IHC	Deep reinforcement learning (DRL)-based model	172 slides in the HER2 scoring contest dataset	The model outperformed other methods based on state-of-the-art deep convolutional networks.
^[48]^	Directly predict HER2 status from HER2 2 + IHC slides.	IHC	Convolutional neural networks with EfficientNet B0 architecture using a transfer learning approach	115 consecutive cases received at the Canadian reference HER2 laboratory	AUC was 0.79 with an overall accuracy of 81% (sensitivity = 0.50, specificity = 0.82) in external validation slides
^[51]^	Scoring and interpreting HER2 IHC	IHC	AI algorithm based on multilayered convolutional neural networks	120 patients from four pathology laboratories in three countries	The AI solution demonstrated high accuracy for HER2 scoring, with 92.1% agreement on slides with high confidence ground truth.
^[56]^	Predicting HER2 status based only on H&E samples	H&E	Models presented by 21 teams worldwide, such as ResNet34, DenseNet201, EfficientNetB0,etc.	359 WSIs from 22 different laboratories	Some models achieving AUC scores above 0.8
^[58]^	Predicting biomarkers based on H&E samples	H&E	Multiple instance deep-learning-based neural network	2535 WSIs from ABCTB dataset and 1014 WSIs from TCGA	AUCs were 0.92, 0.81, and 0.78, for ER, PR, and HER2

Some researches about the application of AI in HER2 assessment of breast cancer are listed in this table.

ABCTB, Australian Breast Cancer Tissue Bank; AIDPATH, Academia and Industry Collaboration for Digital Pathology; AUC, area under curve; DL, deep learning; ER, estrogen receptor; HER2, human epidermal growth factor receptor 2; H&E, hematoxylin and eosin; IHC, immunohistochemistry; PR, progesterone receptor.

The introduction of AI not only reduces the heterogeneity among observers but also enhances the assessment of HER2 status with negative or equivocal expression. Sean *et al*^[48]^ trained a deep learning algorithm to directly predict the HER2 gene amplification status in HER2 2 + slides. This study employed a transfer learning approach to train a CNN with the EfficientNet B0 architecture. Although the performance of this method is not high enough to replace classic FISH testing, it still provides promising results for the further application of AI. Similarly, with the emergence of new HER2-targeted ADCs, the discrimination between HER2 0 and 1 + becomes increasingly crucial and challenging^[49]^. Si Wu *et al*^[50]^ found that the use of AI algorithms can significantly enhance the accuracy and consistency of HER2 0 and 1 + evaluations, particularly in cases of HER2 1 + . But considering that the ROI is manually selected by the pathologist, the final comprehensive score could be influenced by the pathologist’s subjective choice. Meanwhile, another study^[51]^ validated a fully automated AI solution for accurately assisting in scoring HER2 IHC according to the ASCO/CAP 2018/2023 guidelines. The AI solution not only significantly improved the consistency and accuracy of all HER2 scores, but also enhanced the distinction between HER2 0 and HER2 1 + cases. But there are still some specific shortcomings of the algorithm, such as its insufficient performance in distinguishing between HER2 2 + and 1 + cases.

In addition, there are also some studies that have developed models to predict HER2 status from WSIs stained with H&E and demonstrated reliable results^[52–55]^. For example, the HER2 on H&E (HEROHE) challenge, which is the first competition to predict HER2 status in invasive breast cancer samples from H&E-stained WSI, showcased multiple models for predicting HER2 status proposed by twenty-one teams around the world^[56]^. Although some models achieved higher accuracy in HER2 status prediction than previously reported in the literature, such as comprehensibility and interpretability remained be the limitations to be addressed.

Overall, AI models demonstrated higher efficiency and stability, while also consistent results compare with human pathologists in assessing HER2 expression. The ASCO/CAP guidelines have recognized AI algorithms as diagnostic tools for evaluating HER2 IHC scores, and CAP has issued guidelines to promote the application of AI in clinical practice^[57]^.

#### ER and PR

Similar to HER2, there are currently several studies using AI to predict ER/PR expression from pathology images. A study utilized deep learning methods to evaluate the intensity and proportion of ER/PR-immunostained cancer cells in invasive ductal carcinoma and DCIS^[59]^. Another study proposed an AI-driven analyzer for HER2 and ER/PR, consisting of cell and tissue models^[60]^. The analyzer employed DeepLabv3 + segmentation model, with ResNet-34 and ResNet-101 for feature extraction. The results showed that the assistance of AI significantly increased the consistency among three pathologists regarding HER2, ER, and PR status. But FISH test was not available in this study due to the limited number of cases with test results.

In addition, there are also methods developed to predict the expression of hormone receptors based on H&E-stained images. Gil Shamai *et al*^[16]^ developed a ML method called Morphological-Based Molecular Analysis (MBMP). The study found that tissue morphology was significantly associate with the molecular expression of all 19 biomarkers, including ER, PR, and HER2. Another study also indicated that neural networks developed with unannotated datasets can predict HER2, ER, and PR status from H&E images in a large independent test set^[61]^. The limitation of this approach is that they demonstrate its application with respect to ER classification, but not PR or HER2.

However, there are still challenges in using automated AI algorithms to assess ER/PR expression^[62]^. False positive results can arise from the mixing of benign glands in tumors and the DCIS components of invasive cancer. Weak IHC staining that may not be detected by AI algorithms can lead to false negative results.

Overall, there are relatively few AI models specifically developed for ER and PR, while most are comprehensive models for recognizing multiple biomarkers. These models based on IHC could identify the status of HER2, ER and PR simultaneously, which is beneficial for improving the subtype classification of breast cancer. Models developed from H&E slides could significantly reduce the costs and labor which caused by IHC testing.

#### Ki-67

In addition to the classical biomarkers mentioned above, AI has also been used to quantitatively assess other tissue markers, such as Ki-67. Ki-67 is a reliable marker of cell proliferation and elevated Ki-67 expression is associated with poorer prognosis in breast cancer^[63]^. A variety of AI methods, represented by DL, are currently showing strong potential in automated quantification of Ki-67, by increasing the robustness to artefacts and improving the sensitivity of nuclei detection^[64]^. There are already several DL methods developed explicitly for Ki-67 quantification in breast cancer, such as piNet^[65]^ and UV-Net^[64]^. piNET is a novel DL-based proliferation index (PI) calculator for Ki-67, which achieved reliable and accurate results in datasets comprised of patches, tissue microarrays (TMAs) and WSIs, and the PI accuracy rate was 86%^[65]^. UV-Net is an optimized U-Net structure that focused on preserving highresolution details in pathology images for the evaluation of Ki-67^[64]^. Compared to other models, UV-Net achieved higher and consistent performance on multi-center datasets, with an average F1-score of 0.83. Overall, diverse promising AI assessment tools for Ki-67 have been developed with increasingly evident advantages.

### AI in the detection of BRCA mutations

Genetic susceptibility to breast cancer derived from mutations in specific genes, such as the key tumor suppressor gene *BRCA* (*BRCA1* or *BRCA2*). Patients carrying pathogenic mutations in *BRCA1/2* have significantly increased risk of developing breast cancer and may benefit from targeted therapies^[66]^. Currently, the main method for detecting *BRCA* mutations is genetic testing, which is time-consuming and expensive. Therefore, whether AI can be used to directly identify the mutation status of *BRCA* from WSIs is a question worth asking.

Few studies explored the AI application in answering this question. One study utilized the pathological features of H&E-stained WSIs from breast cancer to predict the gBRCA mutations^[67]^. The authors trained a deep CNN based on ResNet on WSIs which were annotated by pathologists and established the gBRCA mutation risk prediction model based on 17 mutants and 47 wilds, which has restricted efficacy and robustness. However, the study still faces the problem of the small number of slides containing BRCA mutation instances that can be used for training and validation. Another study developed a Bi-directional Self-Attention Multiple Instance Learning (BiAMIL) algorithm to detect *BRCA* status from H&E images^[68]^. They established an interpretable deep neural network model based on attention mechanisms to predict the *BRCA* status in breast cancer with satisfied performance (AUC = 0.819), however, no validation was performed, which restrained the credential of the model. In addition, another study employed a publicly available, deep learning-based weakly supervised method to predict the mutation status of *BRCA* in H&E slides of epithelial ovarian cancer. However, the ROC AUC of validation set was only 0.59, which required further improvement^[69]^. Therefore, the AI-based models of detecting *BRCA* mutations in WSIs of breast cancer are still in their infancy, which requires further more research.

### AI in immune-oncology, PD1/PD-L1

Programmed death protein 1 (PD-1) and programmed death ligand-1 (PD-L1) targeted immunotherapy is one of the most promising cancer treatments in recent years, especially in TNBC. The Food and Drug Administration (FDA) has approved the assessment of PD-L1 by Ventana SP142-staining IHC to select candidates for immunotherapy in TNBC patients^[70]^. Given the current lack of standardized scoring methods and the variability in manual scoring, several studies have attempted to utilize AI to directly identify the expression of PD-1 or PD-L1 from digital pathology images.

Gil Shamai *et al*^[71]^ employed state-of-the-art deep learning techniques and constructed a new dataset to evaluate the feasibility of predicting PD-L1 expression in H&E-stained slides in breast cancer. The system developed in this study has consistent predictive performance across two externally validated cohorts. But the limitation of this research was that it was conducted on tissue microarray images that have limited clinical translation, rather than whole slide images. Another study evaluated the expression of PD-L1 by using the open-source digital pathology platform QuPath and found that the results were highly consistent with the manual scoring by pathologists^[72]^. The ability to dynamically identify sensitivity thresholds within this method allows to visually review PD-L1 detection to ensure accurate and robust assessment at these low expression levels.

Despite the promising results demonstrated in these studies, till now only a few AI algorithms for PD-L1 assessment were validated in clinical practice. This may be derived from the complexity of PD-L1 testing, which involves multiple clones of antibodies and distinct scoring systems.

## Could AI conjugate with multi-omics data in the outcome prediction and treatment in breast cancer?

AI advances show significant potential in integrating multi-omics data for breast cancer. Breast cancer, marked by extensive genotypic and phenotypic heterogeneity^[73]^, requires in-depth study of the molecular basis of breast cancer phenotype, development, progression, and metastasis to enable accurate diagnosis, prognosis, and assessment of therapeutic responses^[74]^. The term “omics” encompasses various research areas that explore individual biology at multiple molecular levels, including genomics, transcriptomics, proteomics, and metabolomics, and others^[75]^. Interpretation of multi-omics data could comprehensively capture tumor characteristics, thereby improving precision medicine and guiding personalized treatment.

AI has proven its capacity to analyze complementary multimodal data streams in cancer research. The parallel development of multi-omics technologies and AI algorithms has driven progress in precision medicine for cancer^[76]^. Multi-omics data provide AI with high-dimensional, complex datasets that can be effectively processed through deep learning and ML techniques for diverse purposes, like discovering prognostic biomarkers, identifying predictive biomarkers for treatment in breast cancer^[77]^. Through AI algorithms, researchers can uncover potential biomarkers and demonstrate the ability to integrate multimodal data from existing datasets, identifying new meta-biomarkers and predicting disease progression and recurrence risk^[78]^. AI is particularly advantageous in prognostic modeling for breast cancer, allowing the construction of individualized survival prediction models by integrating multi-scale information from genomic to proteomic data^[79]^.

To date, AI-based tools have achieved substantial success in aiding treatment decision-making, like predicting therapeutic responses and benefits based on multi-omics data of breast cancer^[80]^. For example, Sammut *et al* collected clinical, digital pathology, genomic and transcriptomic data of pre-treatment biopsies of breast tumors and associated these with pathology end points (complete response or residual disease) at surgery^[81]^. These multi-omics features were integrated in predictive model using ML, and the ensemble predictive model achieved an AUC of 0.87 in predicting pCR in an external validation cohort. The results showed that ML models for prediction of therapy response that combine clinical, molecular and digital pathology data significantly outperform those based on clinical variables. In a study by Meti *et al*, researchers evaluated the predictive performance of ML models using clinical and pathological data, comparing them with classic statistical models. They tested multiple ML models – k-nearest neighbor classifier, random forest classifier, naive Bayes algorithm, support vector machine, and multilayer perceptron model – against MLR for predicting response to neoadjuvant chemotherapy (NAC) in breast cancer. The AUC for MLR was 0.64, while among the ML models, the random forest classifier performed best, achieving an AUC of 0.88^[13]^. Another study proposed a hierarchical self-attention-guided deep learning framework to predict NAC response in breast cancer patients using digital histopathology images from pre-treatment biopsy samples. The results indicated that this hierarchical deep learning approach holds strong potential for analyzing pre-treatment tumor biopsy digital pathology images to predict pathological response to NAC in breast cancer (AUC = 0.89)^[25]^. The combined potential of multi-omics, big data, and AI in healthcare is immense. The availability of high-dimensional datasets, along with high-performance computing and innovative ML architectures, would not only increase data utility, but also optimize personalized medicine over single-omics or non-AI studies, bringing the new era of cancer treatment^[82]^.

## The evolution of methodologies of AI in digital pathology of breast cancer

Over the past two decades, technological advances have facilitated the efficient digitization of WSI, leading to a significant growth in the application of AI in digital pathology^[83]^. In early days, Basavanhally *et al*^[84]^ present a computer-aided diagnosis (CAD) scheme to automatically detects and grades the extent of lymphocytic infiltration in digitized HER2 + BC histopathology. Additionally, CAD algorithms are further developed for disease detection, diagnosis, and prognosis prediction to complement the opinion of the pathologist^[85]^.

With the rapid development of computer technology, ML, as a key branch of AI, has gradually permeated into various fields of digital pathology. ML algorithms are able to automatically extract features from a large number of digital slice of images and make predictions based on these learned features, thereby providing a more accurate and efficient tool to aid in diagnosis^[86]^. Supervised learning is a class of ML used for image segmentation^[87–90]^ and disease classification^[91–93]^, such as the segmentation of tumor regions^[90]^ and classification of cardiovascular diseases^[91]^. The other category is unsupervised learning, which performs well in different classification tasks such as benign and malignant tumor detection^[94–97]^ and mass detection in breast cancer^[98]^.

However, as data volumes and the complexity of problems continue to grow, deep learning has emerged as the mainstream choice for analyzing and interpreting histology images^[99]^. DL models include multiple architectures such as CNNs^[12,100]^, GANs^[101]^, and fully convolutional neural networks (FCNNs)^[86]^, the choice of which depends on the particular task being solved. Saltz *et al*^[102]^ described the use of a CNN, combined with feedback from pathologists, to automatically detect the spatial organization of TILs in images of tissue slides from The Cancer Genome Atlas, which could predict the outcome of thirteen types of cancer. Eren *et al*^[103]^ used a U-Net based on FCNN architecture for segmentation of tubules in WSI of breast cancer. Similarly, a Mask R-CNN based on FCNN architecture was employed to detect abnormal lesions, followed by ResNet50 to estimate the malignancy probability in breast cancer^[104]^. In Table 4, we showed the characteristic and application of several representative AI models in digital pathology.

**Table 4:** The application of representative artificial intelligence models in digital pathology

Model	Characteristic	Application
Convolutional neural networks (CNNs)	The CNNs are centred on convolution, and transform the input image by convolutional layers, pooling layers, and fully connected layers, and finally output a class-based likelihood of the image^[89]^.	As the most extensively used DL algorithms, CNN-based approaches have been used for image-based detection and segmentation tasks^[87]^ to identify and quantify cells^[102]^, histological features or regions of interest. These models have achieved excellent results, making CNNs mainstream in classification and segmentation of medical image.
Fully convolutional networks (FCNs)	FCNs replace the fully connected layers in CNNs with convolutional layers, and can accept input images of any size. FCNs can be used to learn representations from every pixel, and therefore are capable of pixel-level predictions, which may have an advantage over CNNs.	U-Net based on FCNs architecture has become probably the most well-known architecture for segmenting medical images^[103]^. Different variants of U-Net, such as V-Net^[107]^, have been proposed and achieved satisfactory results in segmentation performance^[108]^. Mask R-CNN based on FCNs architecture^[104]^ has also achieved good results in segmentation tasks.
Recurrent neural networks (RNNs)	Unlike CNNs and FCNs, RNNs can store previous inputs at different times in order to process them sequentially and apply them to the current output process.	RNN-based AI approach are able to learn patterns from each patch, and generate a patient-level prediction^[109]^. And RNNs can also be used to analyze tissue images obtained at different time points.
Generative adversarial networks (GANs)	GANs consists of two competing neural network models, the generative model generates synthetic data from the input samples, and the discriminative model evaluates the consistency between the generated data and the original data.	Given the advantage of learning hidden data distribution and generating realistic images^[89]^, GAN-based methods are showing increasing promise in DL-based digital pathology approaches, including feature segmentation^[110]^ and stain transfer^[111]^.
Transformers	Transformers are a group of encoder-decoder network architectures used for sequence-to-sequence processing in NLP. Transformers rely on the self-attention mechanisms, which possess the advantage of learning complex, long-range dependencies from input images.	Transformers combined with CNNs can demonstrate better performance in medical image segmentation^[108,112]^. In addition to 2D image segmentation, the hybrid approach is also useful to 3D scenarios^[113]^. Transformers have great potentials due to their strong capability of modeling long-range dependencies.

The characteristic and application of some representative artificial intelligence models in digital pathology are briefly described in this table.

CNN, convolutional neural networks; DL, deep learning; FCN, fully convolutional networks; GAN, generative adversarial networks; NLP, natural language processing; RNN, recurrent neural networks.

Recently, the research frontier in digital pathology has shifted to the integration of multimodal data to meet the need for personalized treatment. Raktim *et al*^[105]^ constructed a deep learning framework that combines image-derived features with genetic and clinical data to obtain a holistic profile and achieve survival risk stratification of ER + breast cancer patients. This model predicted risk (high versus low) with prognostic significance for overall survival in univariate analysis, and maintained independent significance in multivariate analysis incorporating standard clinicopathological variables. Also, there is also the combination of radiology and digital histopathology tissue slides aimed at extracting disease-specific information that is difficult to quantify by the investigators^[106]^.Overall, the application of AI in the digital pathology of breast cancer has been increasingly implemented, which could innovate the multi-omics and model development in the near future, thereby accelerating the era of personalized medicine.

## Conclusion and prospective

**Figure 2.**
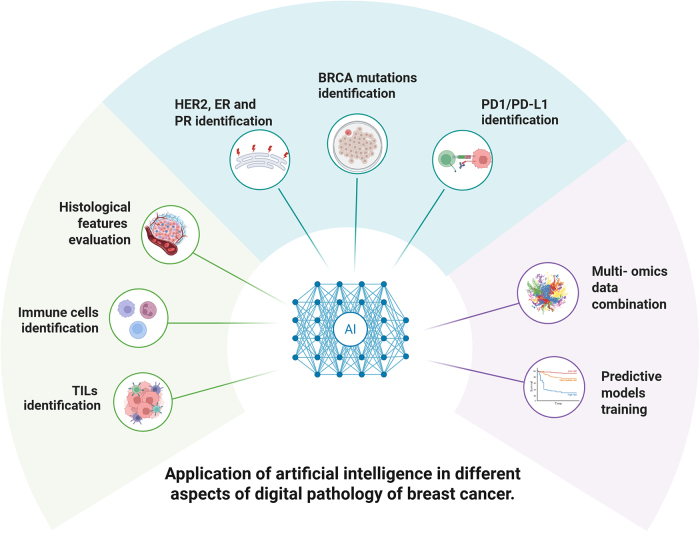
Application of artificial intelligence in different aspects of digital pathology of breast cancer. Artificial intelligence can be used to recognize histological features (such as TIL), classical biomarkers (such as HER2, ER, PR, etc), as well as combine multi-omics data to predict patient outcomes. TME, tumor microenvironment; TIL, tumor-infiltrating lymphocytes; IHC, immunohistochemistry; H&E, hematoxylin and eosin.

In this review, we systematically summarized the current applications of AI in the field of digital pathology of breast cancer, including histological features, classical biomarkers, the combination with multi-omics data, and the evolution of AI techniques. Various AI techniques, represented by deep learning, have been shown consistency with pathologists in identifying and scoring TILs in breast cancer. Also, AI can identify and evaluate the histological features of tumor microenvironment, which could be served as a biomarker of patient outcomes. Furthermore, AI could identify the expression status of classical biomarkers, such as HER2, ER, and PR, from H&E-stained slides, which showed identical findings with classic IHC staining. Lastly, AI could interpret multi-omics data to identify novel biomarkers and to train predictive models in the research of breast cancer (Fig. 2). Therefore, AI has showed robust and promising applications in digital pathology of breast cancer, though more studies are still warrantied.

Currently emerging AI technologies provide new opportunities and directions for refining digital pathology research in breast cancer. The integration of emerging AI technologies can significantly improve the depth of multi-omics analyses and clinical utility. The transformer architecture is a deep learning model that uses a self-attentive mechanism to process input data, and has been used as the core architecture of the underlying model in a number of domains including computer vision, audio processing, time series analysis, and others. By introducing the self-attention mechanism, transformer demonstrates unique advantages in gene sequence feature extraction and the prediction of interactions between histological features^[114]^. Natural language processing (NLP) models based on the transformer architecture can effectively integrate data from the real world (such as patient’s clinical information, lifestyle habits, treatment history, and others) and can significantly improve the accuracy of cancer prognosis prediction^[115]^. Recently, large language models (LLMs) such as GPT-4, LLaMA, deepseek, and Grok 3 have achieved great success and are gradually being applied in areas such as finance, healthcare, and education. These models are capable of efficiently handling complex associations between pathology images and textual data through deep learning and ultra-large-context modeling. The GPT-4 V framework employs in-context learning approach to efficiently classify medical images with a small number of samples without complex model fine-tuning in data-scarce situations^[116]^. At the same time, LLMs models have significantly accelerated the translation process from pathology reports to clinical decisions by parsing millions of medical documents and constructing a pathology knowledge graph^[117]^. These emerging AI technologies offer new possibilities for the development of digital pathology.

In the era of digital healthcare, the research and application of AI in clinical practice is of great significance. AI can assist doctors to improve diagnostic accuracy and consistency, and can reduce human bias. At the same time, automated AI can significantly shorten the time of diagnosis and alleviate the shortage of pathologists, which is especially significant in resource-limited areas. Predictive models with integration of multimodal data (such as genomic, transcriptomic, pathological images, radiological images, and others) can be used to predict patient response to specific therapies, risk stratification and personalized treatment. In addition, AI has powerful data analysis capabilities, which can mine valuable information from complex data set, identify biomarkers that are difficult to find by traditional methods, discover new drug targets, and provide ideas for conducting high-quality clinical research^[118–121]^.

Several clinical trials have already demonstrated the potentiality of AI in clinical settings. A non-randomized, single-centre clinical trial evaluates the effectiveness of an AI-assisted workflow to detect breast cancer metastases in sentinel lymph nodes while maintaining diagnostic safety criteria, and 190 sentinel lymph nodes specimens were consecutively enrolled^[122]^. This study found that with the assistance of AI, pathologists had a significant reduction in adjusted relative risk of IHC use, as well as a significant reduction in time and cost. Another multi-centre clinical trial used a stacking model for accurately predicting axillary lymph node response to NAC using longitudinal MRI in breast cancer. This trial included 1153 patients with node-positive breast cancer who received NAC following surgery and this model showed lower false negative rates compared to radiologists^[123]^. However, most of the researches regarding AI in digital pathology remains in laboratory setting, and most of the clinical trials carried out were on the field of radiology. This indicates that AI methods for pathology assessment is yet mature to applied in clinical practice, and further researches are still warranted.

Although recent studies have showed the potentiality of AI in digital pathology of breast cancer, there are still unavoidable limitations and challenges when conducting the clinical translation of AI. First, the interpretability of AI models may make it difficult to explain the findings and to identify the significant features. Because many ML algorithms are considered as “black box” models, which lack of interpretability and intuitional understanding. It is critical to establish interpretable ML methods for clinical practice. Explainable artificial intelligence (XAI) techniques show promise in bridging the gap between algorithmic innovation and clinical application, such as SHapley Additive exPlanations (SHAP) and gradient-weighted class activation mapping (Grad-CAM). SHAP is a state-of-the-art and all-purpose approach to ML model interpretability, which intuitively quantifies the contribution of each feature to the model predictions and greatly avoids the limitations of the “black box” of traditional models^[124]^. And Grad-CAM provides explainability by visualizing the area on which the model focuses when making predictions in the form of heat maps, intuitively showing the basis used by the model to make decisions^[125]^. The emergence of these approaches offers possible solutions to the obstacles to the clinical application of AI models. Second, another challenge concerning the application of AI algorithms to clinical practice is generalizability of the model. Most AI studies were trained based on small datasets, which may lead to performance degradation when applied to large independent datasets or clinical practice. However, data privacy concerns reduce medical data sharing for model training and limit access to large datasets. Meanwhile, one notable problem is the inherent variability of medical image data, stemming from differences in dyeing methods, scanner resolution, contrast, and signal-to-noise ratios due to diverse clinical protocols. The standardization of medical image to ensure uniformity remains a significant challenge^[126]^. A promising approach to improving the generalization of AI-based models is federated learning, which allows models to securely access sensitive data^[127]^. Federated learning aims to train a ML algorithm collaboratively on multi-institutional data without exchanging them among participating institutions^[128]^. Federated learning can address critical data privacy issues in multi-institutional studies while maintaining model performance and enabling collaborative training of distributed datasets without compromising the confidentiality of private patient data. Although there are also challenges regarding federated learning, this method facilitates learning more generalizable and better-performed algorithms that would better meet the needs of clinical applications. Besides, the accuracy of evaluation of biomarkers like TILs, HER2, ER, etc. from H&E-stained images still warrant further validation. In addition, there are still many other problems when AI is applied to clinical practice. For example, there is still a lack of unified reference standards and regulatory frameworks for AI^[129]^. Most clinical studies are still limited to retrospective analyses with insufficient prospective clinical validation^[130]^. And the lack of digitalisation of pathology workflows and the lack of interpretability of AI may limit patients’ trust in AI diagnosis.

Therefore, AI has made significant progress in the field of digital pathology in breast cancer. Though there are still limitations and challenges, we believe that the fast evolution of AI could tremendously promote the research in digital pathology, thereby declare the coming of new era of practice in precision medicine of breast cancer.

## Data Availability

Not applicable.

## Research registration unique identifying number (UIN)

Review paper, Not applicable.
